# Validation of the French version of the Body Awareness Questionnaire: toward a way to assess alexisomia

**DOI:** 10.3389/fpsyg.2024.1261994

**Published:** 2024-09-06

**Authors:** Arnaud Carre, Rebecca Shankland, Philippe Guillaume, Jeanne Duclos, Claire El-Jor, Sonia Pellissier, Valentin Flaudias

**Affiliations:** ^1^Univ. Savoie Mont Blanc, Univ. Grenoble Alpes, LIP/PC2S, Chambéry, France; ^2^Univ. Lumière Lyon 2, Développement Individu Processus Handicap Éducation, Université Lumière Lyon 2, Lyon, France; ^3^Université Paris 8, Saint-Denis, France; ^4^Univ. Lille 3 Charles de Gaulle, UMR9193 Laboratoires Sciences Cognitives et Sciences Affectives (SCALab), Villeneuve-d'Ascq, France; ^5^Université de Nantes, Nantes, France; ^6^Centre Hospitalier Universitaire de Clermont-Ferrand, Clermont-Ferrand, France

**Keywords:** alexisomia, interoception, body awareness, alexithymia, scale validation

## Abstract

**Background:**

Awareness of one's own states is a particularly important part of cognition and emotion regulation. Recently, the concept of alexisomia has been used to refer to lack of awareness and expression of somatic sensations. Developing self-reported questionnaires to evaluate alexisomia represents a challenge for clinical psychology and medicine. In this context, we suggested to adapt the Body Awareness Questionnaire in French to measure alexisomia and its relation to alexithymia. In fact, we carried out a backtranslation and studied the validity of the construct in relation to proximal constructs around emotional awareness.

**Methods:**

For this study, 610 university students completed questionnaires measuring a three dimensions alexithymia concept [with The Toronto Alexithymia Scale (TAS-20)] or the five dimensions alexithymia concept [with The Bermond-Vorts Alexithymia Questionnaire (BVAQ-B)] and alexisomia (with the BAQ).

**Results:**

Confirmatory factor analyses showed that the BAQ can be envisaged through 4 factors as well as a unidimensional model to refer to alexisomia. We also found that body awareness was negatively related to scores of alexithymia.

**Conclusion:**

Results are discussed in light of the construct of alexisomia and its clinical implications in somatic as well as mental disorders. We suggest that the BAQ, which assesses interoception, can contribute, in part, to the assessment of alexisomia. Like alexithymia, this is a key concept to take into consideration when designing treatment and prevention programs.

## Background

The field of grounded and embodied cognition points to a bidirectional link between emotional processing and body functioning (Barsalou, 2008; Niedenthal et al., 2009). To add to this, the relationship between the perception of a subjective (feeling) and an objective (bodily components) state influences the sense of coherence of the emotional experience (Sze et al., 2010) and appears to play an important role in emotional, behavioral, and health regulation (Mehling et al., 2009). This is important since psychiatric and psychological disorders, as well as Somatic Symptom Disorder, are all related to emotional regulation difficulties (Gross and Jazaieri, 2014). These difficulties occur at the affective, cognitive, and social levels, ranging from emotional awareness to regulatory strategies (Gross, 2002; Barlow et al., 2004; Boden and Thompson, 2015).

In particular, difficulties in emotional awareness, associated with verbalization difficulties, have been widely studied in psychopathology and psychosomatics through the construct of alexithymia (Berthoz et al., 2011; Kojima, 2012; Leweke et al., 2012). Indeed, several meta-analyses have described the significant associations found between alexithymia and different mental health issues such as posttraumatic stress disorder (Frewen et al., 2008), eating disorders (Westwood et al., 2017), schizophrenia (O'Driscoll et al., 2014), self-harm (Norman et al., 2020), and suicidal thoughts and behaviors (Hemming et al., 2019). Additionally, a recent meta-analysis found that alexithymia is prevalent in individuals with Autism Spectrum Disorder but not universally so which means that this specific subgroup may have unique clinical requirements (Kinnaird et al., 2020). Furthermore, another meta-analysis of 77 studies concluded that alexithymia is higher in people with chronic pain with higher reported pain intensity and physical limitations (Aaron et al., 2019). Alexithymia, initially introduced by Sifneos (1973), is used to describe cognitive or personality traits including difficulty identifying emotions and describing feelings to others, externally oriented thinking (a preference for focusing on external events rather than personal feelings), and a limited imaginative capacity (Nemiah et al., 1976). Beyond emotional awareness and its physiological component, greater attention has been paid to the awareness of bodily states. Notably, studies on chronic somatic disorders and pain have led to the proposition that they are characterized by alexithymia associated with physiological hyperarousal and an intense way of perceiving its sensations (and symptoms) as a hypersensitivity (Lumley et al., 1996; Kano and Fukudo, 2013) and sometimes, conversely, a hyposensitivity (Neumann et al., 2004; Rubio et al., 2014). Both situations suggest a mismatch between sensation and perception and reflect a disorder of interoception.

Recently, the clinical importance of impairments in processing somatic sensations has been synthesized (Oka, 2020). Indeed, this helps to understand the lack of awareness of an existent illness and how it can lead to risky behaviors that contribute to maladaptive states (i.e. regulation of behaviors, awareness of fatigue and physical limits, and therapeutical observance). Furthermore, based on the Japanese construct of “*shitsu-taikan-sho*” (“*shitsu*” for lack, “*taikan*” for bodily sensations, and “*sho*” for symptoms), driven by psychosomatic studies, Ikemi suggests impairments that seem to be more extensive than alexithymia (emotion awareness) (Ikemi and Ikemi, 1983). This leads us to the construct of alexisomia, or “no words to describe bodily states,” which is a clinical concept that refers to characteristics of having difficulties in the awareness and expression of somatic sensations (Moriguchi and Komaki, 2013; Kanbara and Fukunaga, 2016). In the most recent evolution of this conceptualization, alexisomia has been viewed as a related but independent construct of alexithymia (Ikemi and Ikemi, 1986). Two recent models of addictive states could highlight the interactions between alexithymia and alexisomia while articulating their differences as cognitive processes (see Noël et al., 2013; Flaudias et al., 2019). The notion of alexithymia is structured within the proposition of a conceptual transfer made by Ikemi and Ikemi (1986) in Oka (2020), who initially sought to transfer the concept of alexithymia into the context of holistic Eastern medicine in general, and Japanese medicine in particular. Authors initially considered alexisomia as a broader problem, independent of alexithymia.

According to some authors, assessments of alexithymia refer to a decrease in interoceptive awareness, and could therefore be a first form of assessment of alexisomia (Brewer et al., 2016). Recently, a scale designed to assess this construct, named the *Shitsu-taikan-sho* Scale (STSS) has been developed and published in English by Oka (2020). This scale is composed of the following three dimensions: (*i)* difficulties in identifying bodily sensations, (*ii)* over-adaptations that refer to excessive responses to social demands which overlooks one's real affective and physical states and needs, and (*iii)* lack of health management based bodily sensations. Alongside the STSS, the Interoception Sensory Questionnaire (ISQ) has been established to assess alexisomatic components in adults with a single factor (Fiene et al., 2018). The ISQ retained 20 items on an initial set of 60 items that encompass several fields that are difficulty in identifying and describing interoceptive bodily signals unless extreme states, the hypo-reactivity and hyper-reactivity, and it also encompasses impairments in affective touch, and a reduction of motivation to manage bodily states.

Nevertheless, at a conceptual level, it appears that non-affective body awareness represents the roots of the concept of alexisomia as it touches on a central deficit of interoception. Moreover, recent research indicates that alexisomia is a way to better understand the processing involved in interoception. As this has been underlined in past research, we suggest measuring alexisomia through a scale that only focuses on this aspect: the Body Awareness Questionnaire (BAQ) (Shields et al., 1989). Indeed, the BAQ calls for explicit vigilance about the variations in body cues and their consequences on health. It is an 18-item scale designed to assess self-reported attention and awareness of normal, non-affective bodily processes. It can be used as a unidimensional perspective of interoception, but it can also be divided into four dimensions. These four dimensions refer to: (*i)* the response or changes in body processes, (*ii)* the prediction of body reaction, (*iii)* the perception of sleep-wake cycles, and (*iv)* the self-prediction of the onset of illness.

Considering that this construct is particularly relevant for the conceptualization of the etiopathogenic process of mental disorders, we are interested in the components and the evaluation of alexisomia. More precisely, awareness of one's internal states goes beyond what alexithymia classically assesses, and we suggest that non-affective bodily awareness constitutes a way of apprehending this “new” large construct of alexisomia. In this sense, we suggest that body awareness is a good candidate for measuring at least a large part of alexisomia, here using the BAQ. With regard to a Western approach, compared to a so-called Eastern approach (and more specifically, according to the literature, one that was developed in Japan), the idea of emotional awareness is revisited here. More specifically, regarding the conceptual approach of self-awareness and awareness of one's internal and external emotional environment, alexithymia appears to be only fragmentary. In other words, it only explains part of the bigger picture. This is where alexisomia comes in, providing a new way of understanding affective and non-affective states. To add to this, Garfinkel and Critchley (2013) suggested that three main dimensions are associated with interoception. The first, called accuracy, refers to behavioral components of interoception, as seen in tasks like heartbeat detection. The second, called sensibility, corresponds to the subjective part of interoceptive processing which, according to these authors, refers to methods such as questionnaires to assess interoception. Finally, the third, which encompasses protocols that include subjective self-report and performance tasks about interoception, is named the metacognitive dimension. As Critchley and Garfinkel (2017) suggested, the dimensions are seen as distinct from each other. Therefore, in order to accurately situate this study in the field of interoception research, we specify that our work corresponds to the subjective aspects called the sensibility dimension (Garfinkel and Critchley, 2013; Critchley and Garfinkel, 2017). This work, through the mobilization of alexithymia alongside interoceptive awareness (whose alteration or problematic functioning refers to this new emerging construct of alexisomia), postulates the continuity of awareness of complementary emotional phenomena: about sensations and affects. This is in line with the proposal made by several studies (Herbert et al., 2011; Moriguchi and Komaki, 2013; Fiene et al., 2018) also suggesting that the two constructs are more closely linked than was initially thought.

Taking all of the above into consideration, the aim of this study was twofold. First, we aimed to validate the French version of the Body Awareness Questionnaire (BAQ) as an assessment of alexisomia functioning, and test its psychometric properties in a general (non-clinical) population. Second, we aimed to test its relation to alexithymia. The relationships between alexithymia and body awareness (that here reflect alexisomia for low scores) contribute to the assessment of convergent validity indicator. To the best of our knowledge, this is the first study that approaches the BAQ through the construct of alexisomia and its relation to alexithymia.

## Methods

### Participants

Six hundred and ten participants that were university students and active workers. All of them agreed to take part in the study. Details of demographic characteristics are shown in Tables 1, 2 for details about levels of education. The study was conducted in three universities (Univ. Grenoble Alpes, Univ. Savoie Mont Blanc, and Univ. Paris 8 Vincennes-Saint-Denis). Participants were included on the primary criterion of being of legal age (at least 18 years old) and giving consent to take part in the study. There were no specific exclusion criteria, except for incomplete data when completing the scales. Participants in the latter group (Univ. Paris 8 Vincennes-Saint-Denis) completed the Toronto Alexithymia Scale (TAS-20) while the others completed the BVAQ to assess alexithymia. Data were acquired in paper-pencil protocols (a part of a sample in one university) as well as by web-designed surveys with no financial compensation. Concerning the assessments online, in all universities, the Limesurvey online survey platform was used. Participants had the opportunity to respond individually under the best conditions chosen by themselves (and not collectively during a university course). Participation was voluntary. Students at one university received an academic bonus for time spent on the study (0.25 points on a single exam graded out of 20 within their semester of study). Seventy-seven participants took part in the re-test 3 weeks later. Participants were aged from 18 to 80 years (Mean age = 30.5, SD =13.2). Level of education was collected by asking “what is your highest level of education.” Answers were collected based on the classification of education levels from the French National Institute for Statistics and Economic Studies (*Institut National de la Statistique et des Etudes Economiques -INSEE*). Academic levels were divided between secondary education and higher education. Due to a technical problem, 168 levels of education were not recorded. Participants were required to complete all scales and only complete data were retained.

**Table 1:** Descriptive statistics.

**Variable**	**Mean**	**SD**	**Females Mean (SD)**	**Males Mean (SD)**	***n* males/*N* total**
Age	30.52	13.21	29.4 (12.6)	34.6 (15.1)	481/610
BAQ-BODY CHANGE	17.6	4.79	17.7 (4.76)	17.1 (4.87)	129/610
BAQ-PRED.BODY	16.4	4.74	16.4 (4.74)	16.6 (4.78)	129/610
BAQ-SLEEP.WAKE	14.1	3.61	14.3 (3.66)	13.7 (3.42)	129/610
BAQ-ILLNESS	14.7	3.78	14.7 (3.82)	14.6 (3.61)	129/610
BAQ-TOTAL	62.8	12.7	63.1 (13)	61.9 (11.6)	129/610
BVAQ-B1	11.5	1.76	11.4 (1.68)	11.8 (1.91)	112/412
BVAQ-B2	9.23	3.33	8.94 (3.17)	9.99 (3.64)	112/412
BVAQ-B3	9.73	2.51	9.59 (2.61)	10.1 (2.22)	112/412
BVAQ-B4	8.91	2.67	8.29 (2.33)	10.6 (2.81)	112/412
BVAQ-B5	7.65	2.51	7.25 (2.25)	8.72 (2.86)	112/412
BVAQ-TOTAL	47	7.59	45.4 (7.04)	51.2 (7.43)	112/412
TAS-20-DIF	16.2	5.52	16.4 (5.48)	14.8 (5.88)	17/198
TAS-20-DDF	12.7	5.19	12.7 (5.26)	12.9 (4.53)	17/198
TAS-20-EOT	15.1	4.16	15 (4.2)	15.6 (3.68)	17/198
TAS-20-Tot	44	11.9	44.1 (12.1)	43.2 (10.4)	17/198

BAQ, Body Awareness Questionnaire; Body.Change, the response or changes in body processes; Pred.Body, the prediction of body reaction; Sleep.Wake, the perception of sleep-wake cycles; Illness, the self-prediction of the onset of illness; BVAQ, Bermond–Vorst Alexithymia Questionnaire; B1, impairments in identifying feelings; B2, impairments in verbalizing feelings; B3, impairments in fantasizing; B4, impairments in emotionalizing; TAS-20, Toronto Alexithymia Scale; DIF, Difficulties in Identifying Feelings; DDF, Difficulties in Describing Feelings; EOT, Externally Oriented Thinking.

**Table 2:** Descriptive levels of education.

**Levels of education**	***N***	**%**
2nd and 3rd cycles (Masters and Doctorate)	133	21.803
Current Undergraduate Degree (in progress)	175	28.689
High-School diploma (USA) or A Levels (UK) (Baccalaur.at in France)	24	3.934
National Vocational Qualification (UK) and Levels before High-School diploma (USA) or A Levels (UK)	21	3.443
Undergraduate Degree (diploma corresponding to 2 or 3 years validated; *diploma called DEUG, BTS, DUT, License* in France)	88	14.426
Missing values	169	27.705

### Procedure

This study was developed in accordance with the Declaration of Helsinki and its later amendments. It was also in accordance with the French Deontological code of Psychology. All participants signed an informed consent before the study and then filled in information about their age, gender and level of education. As this study involved the validation of a tool in the general population, only participants with no significant current comorbidity (psychiatric, somatic or psychological problems assessed by self-report of past and current health issues) and for whom the data were complete were retained.

### Measures

#### Alexisomia: the Body Awareness Questionnaire

The BAQ is an 18-item scale developed by Shields et al. (1989) that assesses consciousness of physical variations, and more precisely attentiveness to non-emotional body functioning. Two different factorial structures have been identified. The first includes four dimensions that encompass body cycles and rhythms, the detection of changes of the body, and the ability to anticipate bodily reactions (Shields et al., 1989). Items are distributed in the following dimensions: items 1, 4, 10, 13, 14, 16 in BAQ-Body Change for “note response or changes in body process” (e.g., *I notice specific body responses to changes in the weather*); items 2, 3, 8, 11, 12, 15, 16 in BAQ-Predict for “predict body reactions” (e.g., *I always know when I've exerted myself to the point where I'll be sore the next day*); items 7, 8, 9, 15, 17, 18 in “sleep–wake cycle” (e.g., *I can accurately predict what time of day lack of sleep will catch up with me*), and items 5, 6, 7, 10 in Illness for “Onset of illness” (e.g., *I know in advance when I'm getting the flu*). The second factorial structure is unidimensional (Lööf et al., 2013). The French version was adapted using the translation and backtranslation process performed by a native English-speaking colleague to ensure its accuracy. Low scores of body awareness reflecting high alexisomia.

#### The Bermond-Vorts Alexithymia Questionnaire

The Bermond-Vorts Alexithymia Questionnaire (BVAQ-B) is a 40-item questionnaire that exists in two parts of 20-items each. We used the BVAQ part B. Participants were asked to read items and select their response on a 5-point Likert scale. The questionnaire comprises five dimensions, which are impairments in describing and/or communicating about emotional reactions (verbalizing, B1), the ability to fantasize about virtual matters (fantasizing, B2), the ability to identify emotions (identifying, B3), the ability to be emotionally aroused (reacting, B4) and the capacity to look for an explanation of emotional reactions (analyzing, B5). High scores reflect high levels of alexithymia (Vorst and Bermond, 2001; de Vroege et al., 2018). A subsample of 412 participants filled the BVAQ. McDonald's ω of reliability of the BVAQ-B was at 0.7*8*.

#### The Toronto Alexithymia Scale

The Toronto Alexithymia Scale (TAS-20) is a 20-item questionnaire where participants are asked to respond on a 5-point Likert scale (Bagby et al., 1994; Loas et al., 1996). High scores reflect high levels of alexithymia. It is composed of three subscales: Difficulties in Identifying Feelings (DIF), Difficulties in Describing Feelings (DDF), and Externally Oriented Thinking (EOT or attention to external events). A subsample of 198 participants filled this scale. McDonald's ω of reliability of the TAS-20 was at 0.86.

The complete database is based on two distinct assessments of alexithymia, motivated by a two-stage collection for which the team of authors chose to have two complementary tools. In fact, the alexithymia construct is based on two distinct and complementary models and measures. This corresponds to two waves of data collection (at different sites). The TAS-20 is the reference tool for alexithymia, based on three dimensions. The BVAQ is a tool whose factorial properties favor a better quality of the alexithymia construct, suggesting five dimensions reflecting the initial conceptual intention (which the TAS-20 failed to do). In a conceptual perspective where a continuum would be envisaged between awareness of sensory states associated with emotions (alexisomia, via BAQ) and affective states associated with emotions (alexithymia, via TAS-20 and BVAQ), we suggest that the tools benefit from being considered together to illustrate this continuum and test their relationship.

### Statistical analyses

In order to determine how many factors to select for the French validation of the scale, we proceeded with the assessment of the dimensionality of the set of items.

First, we verified the distribution of the data. Using the Shapiro–Wilk test, we found that items of the BAQ were not normally distributed (*p*<*0.001* for all items).

We performed an Exploratory Factor Analysis (EFA) and a Confirmatory Factor Analysis (CFA). EFA was used with principal component analysis and oblimin rotation (direct). Factor analysis suitability was examined by the Bartlett test of sphericity and Kaiser-Meye-Olkin (KMO) test of sampling adequacy. CFA was based on a diagonally weighted least squares estimator for ordinal non-normally distributed data in Structural Equation Modeling (Li, 2016; Gana and Broc, 2018). Goodness of fit was evaluated using the χ^2^ test. However, as the χ^2^ test is highly affected by sample size (Bentler and Bonett, 1980), we also considered the relative χ^2^ (ratio χ^2^/Df), which is suggested to be less influenced by the sample size; admissible relative χ^2^ values range from less than two (Ullman, 2001) to less than five (Schumacker and Lomax, 2010). In addition, the following fit indices were assessed: the Comparative Fit Index (CFI; Bentler, 1990), the Tucker-Lewis Index (TLI; Tucker and Lewis, 1973), and the Root Mean Square Error of Approximation (RMSEA; Steiger, 1990). Goodness of fit was indicated by a nonsignificant chi-square test, CFI and TLI values >*0.95* (admissible fit if >*0.90*) and RMSEA < *0.06* (Hu and Bentler, 1999).

Next, internal consistency was assessed using the Cronbach's alpha. Alpha values were interpreted as insufficient when < *0.70*, admissible if between 0.70 and 0.79, good when between 0.80 and 0.89, and values ≥*0.90* were considered as excellent (Cicchetti, 1994). Regarding limitations of the Cronbach's alpha in multidimensional data (Sijtsma, 2009), and taking into account that the BAQ could also be multifactorial, we suggested to proceed with the calculation of McDonald's Omega (McDonald, 2011). After establishing the final factor solution of the scale, we investigated the relation between interoceptive consciousness assessment (BAQ) and external validity indicators, such as alexithymia. Statistical tests were considered significant at *p* < 0.05. Data was analyzed using R version 3.0 (R Core Team, 2023). We used several R packages (“psy,” “psych,” and “lavaan”). During the revision of the paper, additional statistics were done with R studio and JASP software.

## Results

Descriptive statistics of the study are shown in Table 3. Table 4 refers to the analyses of the validity of the French version of the BAQ. Additional data concerning descriptive statistics of the BAQ and frequencies of responses of all items are shown in Table 5. Examination of the percentage of each response modality chosen for each item revealed that no item was systematically associated with one of the extreme choices. In other words, we don't have any ceiling or floor effects that appear with this data (see Table 5).

**Table 3:** Validity of the Body Awareness Questionnaire in French.

**Item**	**McDonald's ω**	**Cronbach's α**	**Kaiser-Meyer's test**	**Mean (SD) [min–max]**
BAQ1	0.835	0.832	0.839	4.552 (1.843) [1–7]
BAQ2	0.836	0.834	0.868	3.725 (1.858) [1–7]
BAQ3	0.835	0.833	0.854	5.120 (1.569) [1–7]
BAQ4	0.826	0.823	0.868	3.661 (1.745) [1–7]
BAQ5	0.835	0.832	0.869	4.743 (1.649) [1–7]
BAQ6	0.836	0.834	0.820	4.721 (1.780) [1–7]
BAQ7	0.834	0.831	0.875	5.230 (1.674) [1–7]
BAQ8	0.833	0.830	0.866	3.936 (1.762) [1–7]
BAQ9	0.832	0.829	0.839	4.548 (1.663) [1–7]
BAQ10	0.846	0.844	0.688	3.044 (1.747) [1–7]
BAQ11	0.829	0.826	0.917	3.769 (1.648) [1–7]
BAQ12	0.833	0.830	0.884	3.790 (1.861) [1–7]
BAQ13	0.837	0.834	0.831	5.118 (1.505) [1–7]
BAQ14	0.837	0.834	0.852	4.272 (1.771) [1–7]
BAQ15	0.829	0.826	0.869	4.179 (1.846) [1–7]
BAQ16	0.826	0.823	0.898	3.652 (1.645) [1–7]
BAQ17	0.838	0.836	0.904	4.469 (1.860) [1–7]
BAQ18	0.834	0.831	0.894	5.115 (1.607) [1–7]
Unidimensional reliability [95% CI]	0.842 [0.823–0.860]	0.839 [0.820–0.857]	0.867 [0.820–0.857]	
Intraclass correlation [95% CI]		0.792 [0.767–0.815]		

The following item was reverse scaled: BAQ10.

BAQ: Body Awareness Questionnaire.

**Table 4:** Correlation analyses.

	**BAQ-body change**	**BAQ-pred.body**	**BAQ-sleep.wake**	**BAQ-illness**	**BAQ-total**
BVAQ-B1	0.057	0.060	0.002	−0.020	0.035
BVAQ-B2	0.035	0.007	−0.123	0.096	0.013
BVAQ-B3	−0.104^*^	−0.195^***^	−0.162^**^	−0.233^***^	−0.216^***^
BVAQ-B4	−0.050	−0.038	−0.006	0.003	−0.056
BVAQ-B5	−0.197^***^	−0.119	−0.225^***^	−0.173^***^	−0.234^***^
BVAQ total	−0.081	−0.120	−0.189^***^	−0.088	−0.141^**^
TAS DIF	−0.060	−0.209^**^	−0.278^***^	−0.209^**^	−0.209^**^
TAS DDF	−0.200^**^	−0.205^**^	−0.300^***^	−0.227^**^	−0.196^**^
TAS EOT	−0.192^**^	−0.100	−0.199^**^	−0.241^***^	−0.222^**^
TAS-20 total	−0.163^*^	−0.214^**^	−0.324^***^	−0.279^***^	−0.247^***^

^*^p < 0.05.

^**^p < 0.01.

^***^p < 0.001.

BAQ, Body Awareness Questionnaire; Body.Change, the response or changes in body processes; Pred.Body, the prediction of body reaction; Sleep.Wake, the perception of sleep-wake cycles; Illness, the self-prediction of the onset of illness; BVAQ, Bermond–Vorst Alexithymia Questionnaire; B1, impairments in identifying feelings; B2, impairments in verbalizing feelings; B3, impairments in fantasizing; B4, impairments in emotionalizing; B5, impairments in analyzing; TAS-20, Toronto Alexithymia Scale; DIF, Difficulties in Identifying Feelings; DDF, Difficulties in Describing Feelings; EOT, Externally Oriented Thinking.

**Table 5:** Frequency table for the items of the Body Awareness Questionnaire.

	**Frequency**	**Percent**	**Cumulative percent**
**BAQ1**			
1	40	6.557	6.557
2	77	12.623	19.180
3	59	9.672	28.852
4	83	13.607	42.459
5	142	23.279	65.738
6	99	16.230	81.967
7	110	18.033	100.000
Total	610	100.000	
**BAQ2**			
1	94	15.410	15.410
2	98	16.066	31.475
3	91	14.918	46.393
4	92	15.082	61.475
5	112	18.361	79.836
6	80	13.115	92.951
7	43	7.049	100.000
Total	610	100.000	
**BAQ3**			
1	20	3.279	3.279
2	30	4.918	8.197
3	50	8.197	16.393
4	66	10.820	27.213
5	156	25.574	52.787
6	167	27.377	80.164
7	121	19.836	100.000
Total	610	100.000	
**BAQ4**			
1	78	12.787	12.787
2	105	17.213	30.000
3	107	17.541	47.541
4	116	19.016	66.557
5	100	16.393	82.951
6	68	11.148	94.098
7	36	5.902	100.000
Total	610	100.000	
**BAQ5**			
1	31	5.082	5.082
2	52	8.525	13.607
3	49	8.033	21.639
4	81	13.279	34.918
5	172	28.197	63.115
6	148	24.262	87.377
7	77	12.623	100.000
Total	610	100.000	
**BAQ6**			
1	37	6.066	6.066
2	54	8.852	14.918
3	64	10.492	25.410
4	80	13.115	38.525
5	134	21.967	60.492
6	134	21.967	82.459
7	107	17.541	100.000
Total	610	100.000	
**BAQ7**			
1	22	3.607	3.607
2	38	6.230	9.836
3	50	8.197	18.033
4	49	8.033	26.066
5	112	18.361	44.426
6	187	30.656	75.082
7	152	24.918	100.000
Total	610	100.000	
**BAQ8**			
1	62	10.164	10.164
2	92	15.082	25.246
3	99	16.230	41.475
4	102	16.721	58.197
5	124	20.328	78.525
6	87	14.262	92.787
7	44	7.213	100.000
Total	610	100.000	
**BAQ9**			
1	37	6.066	6.066
2	44	7.213	13.279
3	76	12.459	25.738
4	113	18.525	44.262
5	139	22.787	67.049
6	133	21.803	88.852
7	68	11.148	100.000
Total	610	100.000	
**BAQ10**			
1	131	21.475	21.475
2	155	25.410	46.885
3	116	19.016	65.902
4	75	12.295	78.197
5	59	9.672	87.869
6	45	7.377	95.246
7	29	4.754	100.000
Total	610	100.000	
**BAQ11**			
1	64	10.492	10.492
2	95	15.574	26.066
3	101	16.557	42.623
4	127	20.820	63.443
5	129	21.148	84.590
6	69	11.311	95.902
7	25	4.098	100.000
Total	610	100.000	
**BAQ12**			
1	90	14.754	14.754
2	101	16.557	31.311
3	81	13.279	44.590
4	87	14.262	58.852
5	111	18.197	77.049
6	106	17.377	94.426
7	34	5.574	100.000
Total	610	100.000	
**BAQ13**			
1	14	2.295	2.295
2	41	6.721	9.016
3	34	5.574	14.590
4	67	10.984	25.574
5	171	28.033	53.607
6	180	29.508	83.115
7	103	16.885	100.000
Total	610	100.000	
**BAQ14**			
1	45	7.377	7.377
2	86	14.098	21.475
3	73	11.967	33.443
4	96	15.738	49.180
5	132	21.639	70.820
6	120	19.672	90.492
7	58	9.508	100.000
Total	610	100.000	
**BAQ15**			
1	55	9.016	9.016
2	97	15.902	24.918
3	72	11.803	36.721
4	84	13.770	50.492
5	126	20.656	71.148
6	114	18.689	89.836
7	62	10.164	100.000
Total	610	100.000	
**BAQ16**			
1	74	12.131	12.131
2	90	14.754	26.885
3	115	18.852	45.738
4	139	22.787	68.525
5	104	17.049	85.574
6	63	10.328	95.902
7	25	4.098	100.000
Total	610	100.000	
**BAQ17**			
1	57	9.344	9.344
2	62	10.164	19.508
3	67	10.984	30.492
4	74	12.131	42.623
5	137	22.459	65.082
6	128	20.984	86.066
7	85	13.934	100.000
Total	610	100.000	
**BAQ18**			
1	21	3.443	3.443
2	36	5.902	9.344
3	46	7.541	16.885
4	66	10.820	27.705
5	146	23.934	51.639
6	170	27.869	79.508
7	125	20.492	100.000
Total	610	100.000	

### Validation of the BAQ

The Kaiser-Maye-Olkin (KMO) revealed good adequation of the sample (*KMO* = *0.867*). This result was sustained for all items (between 0.8*20* and 0.9*17*, except for the 10th item set at 0.6*9*, see Table 3). The Bartlett test of homoscedasticity revealed that analyses could be performed with these data (χ^2^ = *2,478; Df* = *153; p*<*0.001*). Inspection of the scree plot of the parallel analysis including simulated data suggests that the French version of the BAQ can have up to 4 factors (with a high dropout between the first and the second when visualizing eigenvalues).

The results of Structural Equation Modeling (SEM) to process Confirmatory Factor Analyses (CFA) indicated a very good model fit testing the four-factor solution concerning the ratio χ^2^/*Df* (*207.28/124* = *1.671*), and the fit indices [*CFI* = *0.985; TLI* = *0.978; RMSEA* = *0.033 (CI*_90_ = *0.025–0.041)]*. Regarding the standard loadings of items, all were >0.30 except items number 7 (0.2*0*), 10 (0.2*3*), and 15 (0.2*0*). None of these items was concerned by the study of Modification Indices, so we decided to test a model without these items. After removing them, the model was not better than the previous one: χ^2^/*Df* (*149.01/98* = *1.52*), *CFI* = *0.987; TLI* = *0.984; RMSEA* = *0.029 (CI*_90_ = *0.019–0.038)*.

**Figure 1:**
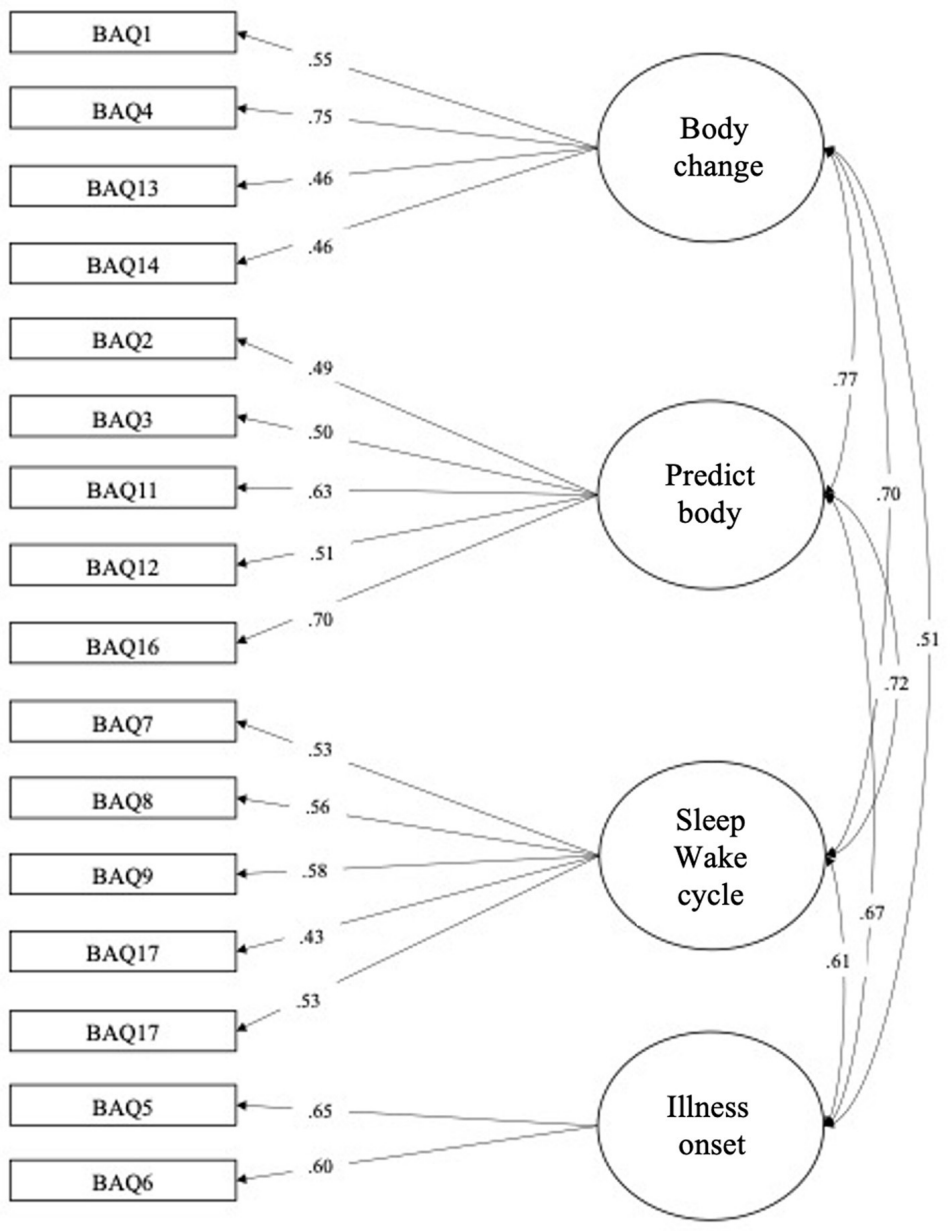
Confirmatory factorial analysis. Four-factors solution of the Body Awareness Questionnaire (BAQ).

**Figure 2:**
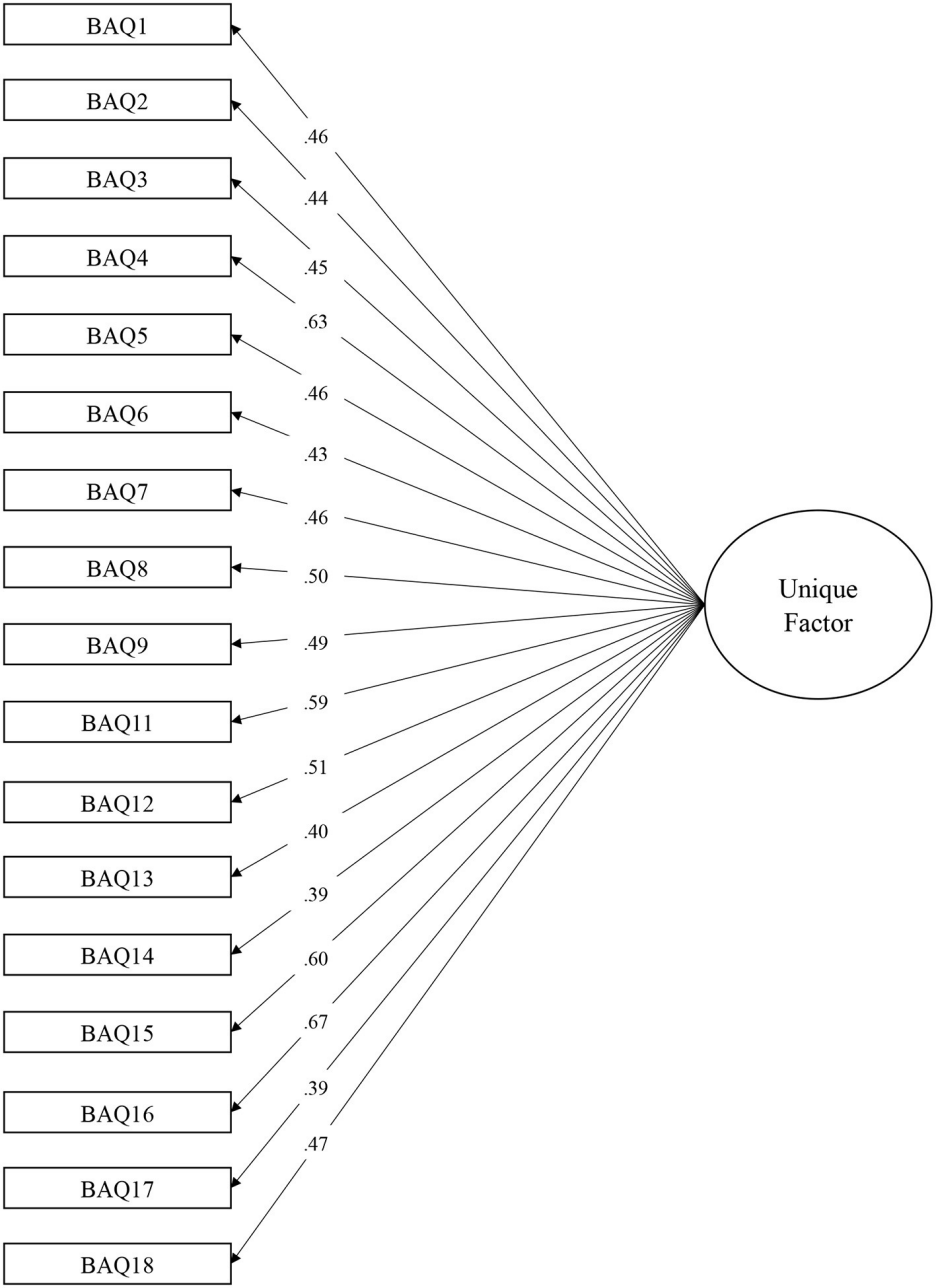
Confirmatory factorial analysis. Final undimensional model of the Body Awareness Questionnaire (BAQ).

The unique factor solution of the BAQ revealed acceptable but worse fits concerning the ratio χ^2^/*Df* (*316.713/135* = *2.346*), and the fit indices [*CFI* = *0.961; TLI* = *0.956; RMSEA* = *0.047 (CI*_90_ = *0.040–0.054)*]. Mc Donald's ω of reliability was at 0.8*39*, and Cronbach's α was set at 0.8*22*. In comparison, the Cronbach's α of the original scale in English (Shields et al., 1989) was set between 0.8*1* and 0.8*2*. Comparison of these two models (four factors vs. one factor) showed that the unidimensional model was better (Δ χ^2^ = *132.16; p*<*0.001*). Figures 1, 2 showed factorial structures of the final model tested with 1 or 4 factors.

### Psychometric properties of the BAQ

Interoceptive consciousness (BAQ total score) negatively correlated with difficulties in identifying emotions (BVAQ-B3 identifying emotions, *r* = −0.22, *p* < 0.001 and TAS-DIF Difficulties in Identifying Feelings, *r* = −0.21, *p* < 0.01), and difficulties in analyzing emotions (BVAQ-B5 analyzing, *r* = −0.23, *p* < 0.001 and TAS-EOT Externally Oriented Thinking, *r* = −0.22, *p* < 0.001). It was also negatively related to difficulties in describing feelings (TAS-DDF, *r* = −0.20, *p* < 0.01).

Regarding correlations between the four subdimensions of the BAQ and the various dimensions of alexithymia (assessed by the two scales), we found significant negative correlations with the BVAQ-B3 (identifying emotions) and the BVAQ-B5 (analyzing). Specifically, the BVAQ-B3 subdimension was significantly negatively correlated with the following subscales of the BAQ: body changes, *r* = −0.10, *p* < 0.05; predict body reactions, *r* = −0.20, *p* < 0.001; sleep wake cycles, *r* = −0.16, *p* < 0.01; onset of illness, *r* = −0.23, *p* < 0.001. Concerning the BVAQ-B5 subdimension, it was significantly negatively correlated with the following subscales of the BAQ: body change, *r* = −0.20, *p* < 0.001; sleep wake cycles, *r* = −0.23, *p* < 0.001; onset of illness, *r* = −0.70, *p* < 0.001, respectively.

**Figure 3:**
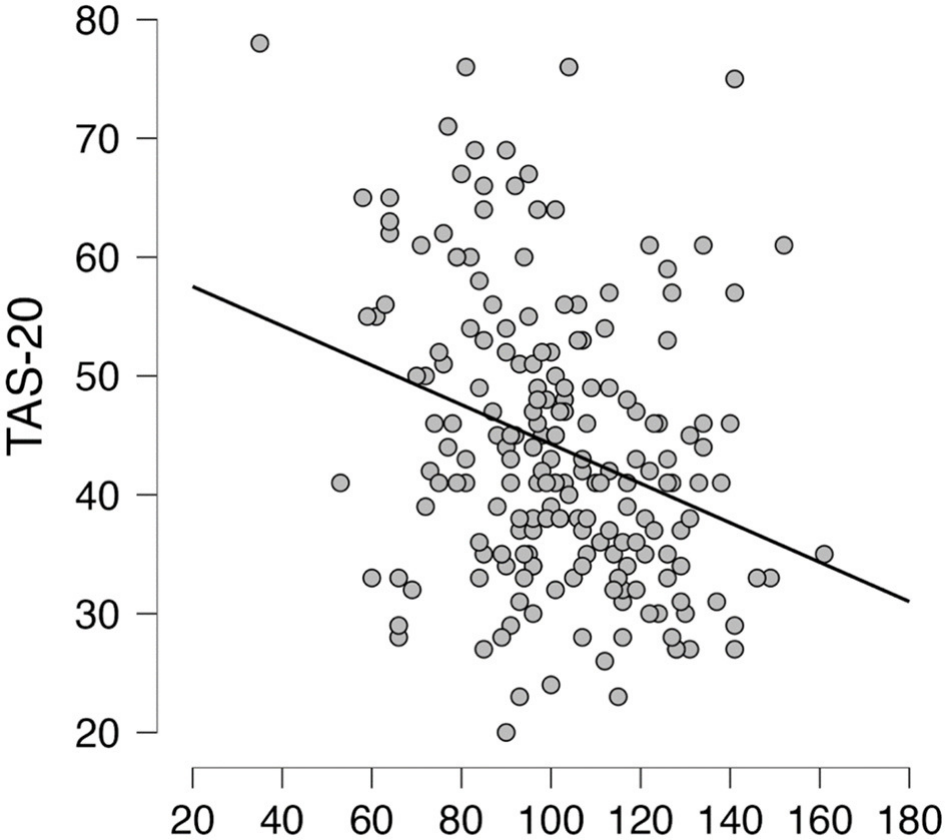
Correlation analysis. Scatterplot between Toronto Alexithymia Scale (TAS-20) and the Body Awareness Questionnaire (BAQ).

**Figure 4:**
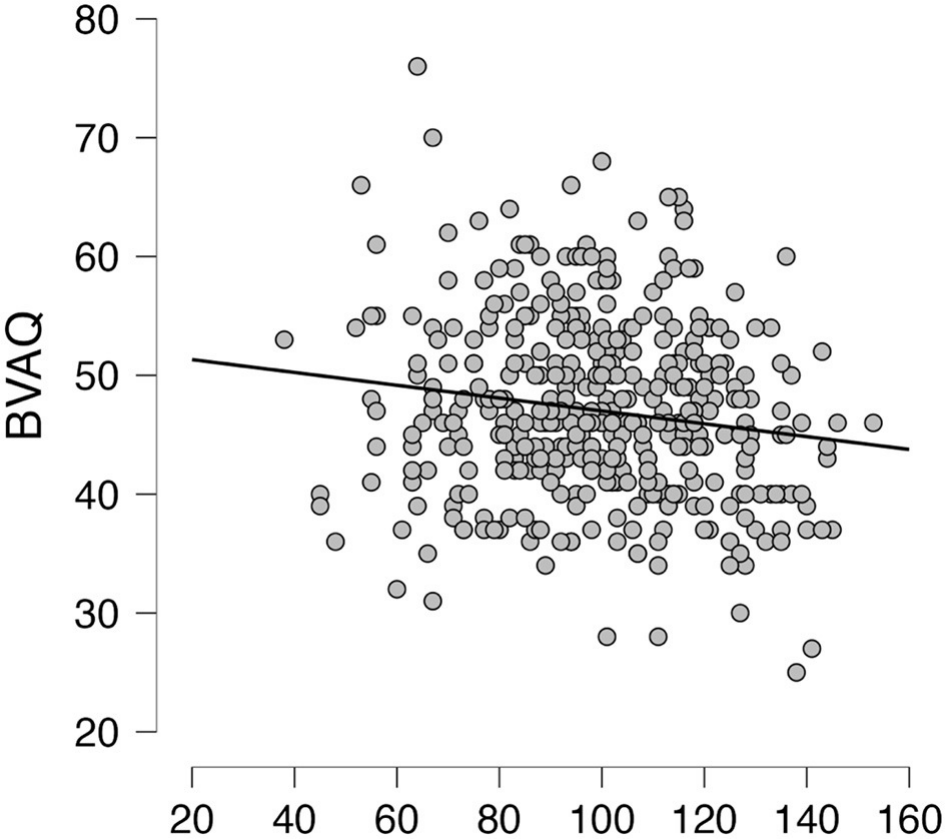
Correlation analysis. Scatterplot between the Bermond-Vorst Alexithymia Questionnaire (BVAQ) and the Body Awareness Questionnaire (BAQ).

Subdimensions of the BAQ concerning the awareness of sleep-wake cycles (BAQ-SLEEP.WAKE) and the onset of illness (BAQ-ILLNESS) were both related to alexithymia as assessed by the TAS-20. Namely, the sleep wake cycles subdimension of the BAQ was significantly negatively correlated with difficulties in identifying feelings (*r* = −0.28, *p* < 0.001), difficulties in describing feelings (*r* = −0.30, *p* < 0.001), externally oriented thinking (*r* = −0.20, *p* < 0.01), and the total TAS-20 score (*r* = −0.32, *p* < 0.001). Similarly, the onset of illness subdimension was significantly negatively correlated with the all subscales of the TAS-20 and the total score: *r* = −0.21, *p* < 0.01; *r* = −0.23, *p* < 0.01; *r* = −0.23, *p* < 0.001; *r* = −0.24, *p* < 0.001, and *r* = −0.28, *p* < 0.001, respectively. All correlations can be found in Table 4. Figures 3 and 4 show the correlations between the scales.

Test and retest correlations were generated based on an interval of 3 weeks. Total scores showed a high temporal reliability (*r* = *0.81; p*<*0.001*). The response or changes in body processes was 0.7*3* (*p*<*0.001*); the prediction of body reaction correlation was 0.7*1* (*p*<*0.001*); the perception of sleep-wake cycles was 0.88 (*p* < 0.001), and finally the self-prediction of the onset of illness was 0.7*1* (*p*<*0.001*).

## Discussion

The goal of the present study was to investigate the psychometric properties of the French version of the Body Awareness Questionnaire (BAQ) in a sample of healthy participants in order to study the construct of alexisomia and its links to the various dimensions of alexithymia in a non-clinical population. To our knowledge, this was the first time that the BAQ was adapted and validated in French.

Considering the elements of the literature regarding the importance of body awareness, and more specifically of the construct of alexisomia, we expected that the assessment proposed by the BAQ would largely reflect the concept of alexisomia. This includes what might be considered a first level of alexisomia, through difficulty in identifying bodily sensations, and difficulty in caring for one's health on the basis of bodily signals.

Our results showed admissible internal consistency and reliability of this translated scale. Based on previous validation studies, we expected that the BAQ could present a good fit with either the four or the one factor model. Confirmatory Factorial Analysis (CFA) revealed that a modified four-factor model correctly fit the data thus supporting the initial factorial structure of the BAQ (Shields et al., 1989) for use in the general population of French adults.

Regarding correlations between the three scales, we found that scores of body awareness were negatively related to scores of alexithymia. This was especially the case for the subscales that rely on identifying and analyzing emotions. Specifically, in line with Critchley and Garfinkel's (2017) sensibility dimension, it appears that the weakest capacities to be connected to one's non-affective internal states are associated with weaker capacities to be in connection with more elaborate mechanisms of awareness of one's own affective states. This is seen in the low BAQ scores that reflect part of alexisomia. It is particularly interesting to note that this potential impact of alexisomia on alexithymia concerns only cognitive components of alexithymia but not affective components. Indeed, our results showed only significant low or moderate correlations between awareness of bodily and affective states, emphasizing both the relatedness and the distinction between alexithymia and alexisomia. In other words, while the negative correlations between the BAQ and the TAS-20 subscales that involve affective states were statistically significant, the highest correlation coefficient was 0.32 (a “low” correlation ranges from 0 to 0.3). This result is in line with a recent study investigating the two constructs and detailing the kind of relationships that could exist (Oka, 2020). To add to this, with our results in mind, it can be argued that the BVAQ has a more sophisticated factor structure than the TAS-20 in terms of its number of factors and its agreement with the theoretical model of alexithymia since it not only assesses difficulties in identifying and describing emotions in addition to the tendency to focus on external events but also difficulties in analyzing and reacting to emotions as well as the ability to fantasize. Additionally, the TAS-20 seems to indicate a consistent negative association of alexithymia with body awareness (with its three factors DIF, DDF and EOT).

A key message of this paper is that the BAQ can be considered as a self-assessment of alexisomia. However, using the BAQ in this manner is not without limitations, in particular, regarding the understanding of the articulation between the different components of alexisomia, and the articulation between alexisomia and other markers of lack of emotional awareness (i.e., alexithymia) or even behavioral aspects, and interpersonal emotional processes (i.e., empathy).

Recent research has pointed out an independent factor within the TAS-20 that reflects interoceptive abilities and is associated with more somatic disorders and medication intake (Fournier et al., 2019). Furthermore, a recent review (Pinna et al., 2020) identified the importance of alexithymia in the response to treatment in psychiatry. Our results are in line with these studies and are consistent with previous knowledge indicating that patients with a psychosomatic disorder with high levels of alexithymia show poorer interoceptive accuracy (Bogaerts et al., 2008). Indeed, health appears to be associated with both emotional and somatic regulatory and homeostatic processes (Tsakiris and Critchley, 2016) and thus intimately related to interoception (Bonaz et al., 2021). Therefore, impairments represent a path to somatic or mental disorders, as seen in alexithymia (Kojima, 2012). This highlights the important link between body perceptions and emotions in wellbeing and prevention of mental health disorders as shown in gastrointestinal chronic diseases where alexithymia-related interoceptive difficulties are significant risk factors (Fournier et al., 2019). Our results further underline this important relationship.

We are in full agreement with Moriguchi and Komaki's (2013) major theoretical contribution, which deals with the multilevel approach to disordered emotional regulation. Taking up this conception, it is possible to conceive a spectrum of emotional impairments ranging from alexisomia to alexithymia. At the most basic levels, i.e. the physiological and primitive aspect of emotional awareness, based on the central interoceptive aspects of the process, it would be this component specifically that would refer to alexisomia. At the most elaborate levels of mental conceptualization of the phenomenon or process of emotional information processing, there is an ability to label. This approach makes it possible to avoid restricting alexithymia to a general deficit in interoception, and also to avoid reducing the interoceptive deficit to the single construct of alexithymia, which we believe would have difficulty in representing the totality of a mechanism. Alexisomia thus makes it possible to model part of the problem of emotional regulation, and more specifically that concerning the disruption of adequate processing of somatic signals. In psychological disorders such as panic disorders, or borderline personality disorders, we support the hypothesis that hyperfocusing on physical sensations without being able to clearly process (or mentalize and regulate) them would lead to an inadequate processing of physiological experience (ie. highly unpleasant, aversive) combined with incomplete mental experience (i.e. lack of global comprehension and mentalization, associated and feedback), which may lead to maladaptive behaviors (i.e. avoidance, risk-taking) aimed at regulating this inner experience. Based on the literature, let us clarify again that the relationship between alexithymia and interoception (here represented in its impaired facet via the construct of alexisomia) is considered by the fact that altered feelings, as well as discomfort or even distress, can be consciously noticed by the individual while other individuals remain unaware. This lack of awareness is partly covered by the construct of alexithymia in the case of emotions and by the concept of alexisomia in the case of sensations (Oka, 2020).

In a clear perspective where alexithymia does not allow to account for all of the disorders in emotional consciousness, and where it would be necessary to specify the levels of alteration of consciousness of one's own states, we formulate the following definitions in in line with the Ikemi and collaborators, (1986). Thus, alexithymia would refer to a difficulty in putting affective states into words (“no words for emotions”), supposedly corresponding at a neuropsychological level to a dissociation of cognitive functioning supported by neocortical complexes and primary emotional (sensory) networks supported by subcortical complexes. Alexisomia would correspond to a disturbance in the identification and verbalization of sensations (“no words for sensations”). This characteristic would be mainly for non-affective consciousness (sensations associated or not with emotions). Alexisomia is generally about the awareness of non-affective bodily sensations, which gives this dimension a fundamental explanatory role in interoception deficits. It is because of this link that we postulated that the BAQ would be able to represent alexisomia. According to Ikemi and Ikemi (1986), alexisomia is a characteristic mainly associated with alexithymia in many patients. If alexithymia and alexisomia have been postulated to indicate difficulties in feeling and expressing emotional and bodily states, another conception may also emerge: alexicosmia. This concept involves a difficulty in awareness and emotional verbalization, which has been described as the lack of connecting to nature and environment, and the lack of awareness of the natural order (Oka, 2020).

### Perspective and further studies

One of the perspectives of this work, which supports the emergence of the alexisomia construct alongside that of alexithymia, is to gain greater precision and sensitivity on the defects of perception of one's own states. The aim here is to consider a differential approach, distinguishing alexithymia for emotional states and alexisomia for bodily states, and the relationship that may exist between the two. Knowing the domain of lack of awareness (i.e. interoceptive or emotional) will be useful in proposing appropriate strategies, suggesting that alexithymia is sometimes used as too broad a construct. Taking charge of the holistic, interoceptive and affective components appears to be a promising and effective approach. In the context of endocrinology, like diabetes (particularly type 1) or central adrenocortical insufficiency, individuals sometimes experience difficulties in emotional regulation in states of high glycemic variation (e.g. hypoglycemia associated with a form of hyperactivity, or hyperglycemia associated with high levels of anger), while at the same time having difficulty in perceiving the actual state and the consequences of their low or high glycemia (Yamashita and Matsubayashi, 2023). Working on alexisomatic characteristics of psychopathological patients appears to be relevant before working on emotional states as assumed by mind-body therapies (Cerritelli et al., 2021) and body-oriented psychotherapies (Galbusera et al., 2019). Therefore, we propose that approaching body awareness from a neutral point of view, that is to say without affect-related items and constructs, could act as an initial step in therapeutical interventions, representing a first access to internal states before more complex representations based on emotions. The reasoning behind this, following a neuropsychological model of emotional awareness (Moriguchi and Komaki, 2013), is that the awareness of one's bodily sensations (that is to say, treating alexisomia) would enable patients to better succeed in becoming more finely and explicitly aware of their own emotional states (treating alexithymia).

Although we conducted correlations between two tools to collect self-reports related to the constructs of alexisomia and alexithymia, the fundamental links between the constructs must be considered with caution. In the case of alexithymic individuals who do not recognize that bodily sensations of emotion are due to emotional phenomena, bodily sensations are frequently interpreted as indicative of a medical illness generated by increased somatization. Additionally, a sufficient level of emotional awareness, going beyond perceptual processing to insert an integrative and representational aspect, is likely impacted in alexithymia (Smith et al., 2018). This leaves the question as to whether alexisomia is fundamentally independent of unresolved alexithymia. Complementary studies, both at fundamental and clinical levels, using dedicated methodological approaches are necessary to clearly understand the partial or total implications, and through this, the different relationships or independence that are questioned in the link between these two constructs.

In addition, it is important to have French validations of other tools measuring interoceptive awareness and alexisomia. Specifically, it appears that further studies are needed on the convergent validity of interoceptive awareness with the BAQ, and more generally about alexisomia in French. This constitutes a limitation of the present study that needs to be addressed. Thus, we propose that it would be important to test the relationship between interoceptive awareness and alexisomia on the one hand, and global elements of psychopathology, such as anxiety and depression on the other. This would strengthen the understanding of both convergent and divergent validity in addition to making it possible to compare several constructs associated with psychopathological functioning and to ensure a specificity of body consciousness.

With that in mind, the psychometric relations assessed by questionnaires should be replicated in the general population, as well as in clinical populations. Investigating clinical samples from somatic and/or psychiatric disorders, including pain, eating, and addictive disorders, could be informative about the way affective and non-affective interoceptive processes are impaired. Moreover, studies using other methodologies, such as experimental or neurophysiological protocols to assess these constructs and the relationship between them should be conducted in a perspective of a multilevel and comprehensive view of mechanisms. A limitation to be addressed in future studies would be to make clear use of “modern preregistration practices,” particularly for testing clinical hypotheses and by proposing *a priori* statistical power calculations. This will make future studies in the fields of interoceptive awareness and alexisomia more robust.

Finally, the appropriate assessment of alexisomia has many implications in the field of somatic and mental disorders. In particular, psychological interventions can be envisaged around problems of regulation of emotions which integrate disturbances in interoceptive awareness. For example, in the context of eating disorders, the alexisomia component remains to be investigated in the light of this construct, but it is well established that the processing of bodily information is altered and that it can be the object of remediation through the practice of mindfulness (Hölzel et al., 2011; Shankland et al., 2016). Additionally, impairments in interoceptive processes related to craving in addictive disorders lead to better understanding patients' alexisomatic characteristics (Flaudias et al., 2019). In clinical contexts, the assessment of alexisomia (associated with other impaired components of emotional regulation) represents a way of better understanding specific dysfunctions of individuals and evaluating the effects of treatment. This can allow for better conceptualization of difficulties related to interoception and to design psychotherapeutic treatments based on the patient's characteristics. Behavioral, cognitive, and emotional therapies can thus focus on the access to bodily awareness and its processing with affective states. We suggest that this could foster new learning thus helping to reduce mental health disorders.

## Conclusion

To the best of our knowledge, this is one of the few Western articles (in Europe) to address this construct of alexisomia. Alexisomia is a concept which has recently raised the attention of researchers and clinicians in the same way as alexithymia has been shown to be particularly useful to design treatment and prevention programs. Focusing on interoception processing and impairments could be a means of better understanding emotions (Garfinkel and Critchley, 2013; Critchley and Garfinkel, 2017) and would be a relevant target for psychotherapies. This article highlights the relationship between dimensions of body awareness and emotional awareness. These relationships are derived from a non-clinical, healthy general population. The work on the awareness of bodily sensations refers in its deficit conception to alexisomia. Our purpose here is to focus on this important and underrepresented construct, alexisomia, and the BAQ can be considered as a way to assess it. We propose that this work is a useful step in the direction of quantifying alexisomia in relation to alexithymia as our study suggests that both concepts are related but distinct. Further studies may test how reducing alexisomia can be a first step to reducing alexithymia in general as well as in clinical populations. This would help improve knowledge about the main psychological mechanisms which help develop emotional competences and hence improve mental health.

## Data availability statement

The datasets presented in this study can be found in online repositories. The names of the repository/repositories and accession number(s) can be found at: https://osf.io/73bs8/.

## Ethics statement

Ethical review and approval was not required for the study on human participants in accordance with the local legislation and institutional requirements. The studies were conducted in accordance with the local legislation and institutional requirements. The participants provided their written informed consent to participate in this study.

## Author contributions

AC: Writing – review & editing, Writing – original draft, Project administration, Methodology, Investigation, Formal analysis, Conceptualization. RS: Writing – review & editing, Writing – original draft, Supervision, Resources, Investigation, Funding acquisition, Data curation, Conceptualization. PG: Writing – original draft, Methodology, Data curation, Conceptualization. JD: Writing – original draft, Conceptualization. CE-J: Writing – review & editing, Writing – original draft. SP: Writing – original draft, Supervision, Methodology, Conceptualization. VF: Writing – review & editing, Writing – original draft, Supervision, Formal analysis, Conceptualization.
